# Spatial patterns of coral survivorship: impacts of adult proximity versus other drivers of localized mortality

**DOI:** 10.7717/peerj.1440

**Published:** 2015-11-24

**Authors:** David A. Gibbs, Mark E. Hay

**Affiliations:** 1 School of Biology, Georgia Institute of Technology, Atlanta, GA, United States; 2 School of Biology and Aquatic Chemical Ecology Center, Georgia Institute of Technology, Atlanta, GA, United States; 3 Current affiliation: Tetra Tech, Inc., Atlanta, GA, United States

**Keywords:** *Pocillopora*, *Seriatopora*, Janzen–Connell hypothesis, *Balistapus*, Predation

## Abstract

Species-specific enemies may promote prey coexistence through negative distance- and density-dependent survival of juveniles near conspecific adults. We tested this mechanism by transplanting juvenile-sized fragments of the brooding corals *Pocillopora damicornis* and *Seriatopora hystrix* 3, 12, 24 and 182 cm up- and down-current of conspecific adults and monitoring their survival and condition over time. We also characterized the spatial distribution of *P. damicornis* and *S. hystrix* within replicate plots on three Fijian reef flats and measured the distribution of small colonies within 2 m of larger colonies of each species. Juvenile-sized transplants exhibited no differences in survivorship as a function of distance from adult *P. damicornis* or *S. hystrix*. Additionally, both *P. damicornis* and *S. hystrix* were aggregated rather than overdispersed on natural reefs. However, a pattern of juveniles being aggregated near adults while larger (and probably older) colonies were not suggests that greater mortality near large adults could occur over longer periods of time or that size-dependent mortality was occurring. While we found minimal evidence of greater mortality of small colonies near adult conspecifics in our transplant experiments, we did document hot-spots of species-specific corallivory. We detected spatially localized and temporally persistent predation on *P. damicornis* by the territorial triggerfish *Balistapus undulatus*. This patchy predation did not occur for *S. hystrix*. This variable selective regime in an otherwise more uniform environment could be one mechanism maintaining diversity of corals on Indo-Pacific reefs.

## Introduction

The processes maintaining high numbers of species in tropical rainforests and coral reefs have long been investigated (Connell, 1978). One suggested mechanism for maintaining diversity is the Janzen–Connell hypothesis (Janzen, 1970; Connell, 1971), which proposes that species-specific enemies clustered near adults increase the local mortality of conspecific juveniles and prevent any single species from monopolizing resources. It has generally been applied to long-lived, stationary, terrestrial organisms such as trees (Zhu et al., 2013). Although there are examples of species-specific distance- or density-dependent mortality affecting community species richness (e.g., Packer & Clay, 2000; Petermann et al., 2008; Bagchi et al., 2014), a meta-analysis found no general, net effect of distance from parent on offspring mortality across a variety of plant types, habitats, or life stages (Hyatt et al., 2003). Thus, some tree species may experience Janzen–Connell effects (Johnson et al., 2012) but the generality of the pattern has been difficult to document (Hyatt et al., 2003). In part, this may be because numerous other processes (habitat heterogeneity, spatial patterns of competitors, etc.) could obscure Janzen–Connell effects. This makes experimental tests difficult in field settings, especially when spatial scales over which they may be relevant are unclear.

Research addressing Janzen–Connell effects on coral reefs is rare (Marhaver et al., 2013). Explanations for maintenance of coral diversity more often invoke disturbance regimes, abiotic gradients (e.g., light, sedimentation), and competition hierarchies (Lang, 1973; Connell, 1978; Buss & Jackson, 1979; Porter et al., 1981). One reason for the paucity of tests in marine systems may be that the hypothesis assumes that dispersal decreases monotonically with distance from parents and that the average dispersal distance is greater than the average distance from the parent at which predation occurs but that they are on the same order of magnitude (Nathan & Casagrandi, 2004), neither of which necessarily applies to marine species with pelagic larvae. Coral larvae are competent to settle within hours of release to months after release (Richmond, 1987; Miller & Mundy, 2003; Nozawa & Harrison, 2008) and may disperse up to hundreds of kilometers (Jones et al., 2009; Torda et al., 2013). Therefore, unlike seeds of many tree species, coral larvae need not be distributed as “seed shadows” with juveniles clustered near parents.

Nevertheless, distance- or density-dependent mortality of juveniles could affect coral species employing either of the sexual reproduction methods that corals use: broadcast spawning, in which eggs and sperm are released into the water column and fertilized eggs develop outside corals, or brooding, in which sperm are released into the water column and fertilize eggs retained inside adult coral colonies. Larvae produced by both methods select their settlement sites and can be attracted to the chemical cues of conspecifics (Dixson, Abrego & Hay, 2014); this could lead to larvae settling near conspecific adults or in aggregations (e.g., Dunstan & Johnson, 1998). Additionally, brooding corals may cast larval shadows akin to terrestrial seed shadows because larvae from brooding corals frequently settle quickly and close to their parents (Carlon & Olson, 1993; Tioho, Tokeshi & Nojima, 2001; Vermeij, 2005; Vermeij & Sandin, 2008; Torda et al., 2013).

Selective mortality of juveniles near conspecific adults is often assumed to be due to specialist enemies that accumulate near adults over their lifetimes. While this may be the case for terrestrial plants where many herbivores and pests are specialists (Bernays, 1989), it is unclear to what extent this applies to corals, of which there are relatively few identified species-specific consumers that might be expected to accumulate near adults of specific prey species (Cornell & Karlson, 2000; Rotjan & Lewis, 2008; but see Neudecker, 1979 and Jayewardene, Donahue & Birkeland, 2009 for examples of coral-specific predation).

However, there is growing evidence from both terrestrial (Packer & Clay, 2000; Bagchi et al., 2014; Fricke, Tewksbury & Rogers, 2014) and marine systems (Marhaver et al., 2013) that microbial pathogens may accumulate near adults and suppress the survivorship of conspecific recruits or juveniles. In the most direct test of the Janzen–Connell hypothesis in corals, Marhaver et al. (2013) used a series of lab and field investigations in the Caribbean to attribute higher mortality of *Orbicella* (formerly *Montastraea*) *faveolata* recruits placed near adult conspecifics to adult-associated microbial enemies. They found a complex relationship between distance from adult colonies, current direction, and recruit mortality. In less direct tests, Vermeij (2005) and Vermeij & Sandin (2008) observed that survival of coral recruits decreased with increasing cover of conspecifics; they hypothesized that this was due to species-specific microorganisms rather than to saturation of a limiting resource.

No study has explicitly tested the Janzen–Connell hypothesis in brooding corals or in fragments typical of juvenile-sized corals. These conditions may generate different results from previously tested conditions because brooding corals may be subject to different distance-dependent mortality patterns compared to broadcast spawning corals and because small fragments may differ from newly settled larvae. For instance, distance-dependent mortality is known to affect some seedlings more than seeds in terrestrial systems (Hyatt et al., 2003). We experimentally evaluated distance-dependent mortality of juvenile-sized corals in the field and correlatively surveyed multiple reefs for patterns of spatial distribution suggestive of Janzen–Connell effects. We focused on two brooding coral species (*Seriatopora hystrix* and *Pocillopora damicornis*) whose planulae recruit over short distances, the latter of which is known to be a preferred prey for some coral consumers (Neudecker, 1979).

## Methods

### Study site characteristics

This study was conducted on reef flats within no-take marine protected areas (MPAs) adjacent to Votua, Vatu-o-lailai, and Namada villages along the Coral Coast of Viti Levu, Fiji. These reserves are scattered along 11 km of fringing reef and are separated by ∼3–8 km. The reserves have high coral cover (38–56%), low macroalgal cover (1–3%), and a high biomass and diversity of herbivorous fishes (Rasher, Hoey & Hay, 2013; Bonaldo & Hay, 2014). The reef flats range from ∼1–3 m deep at high tide, exposed to ∼1.5 m deep at low tide, extend ∼500–600 m from shore to the reef crest, and are typical of exposed reef flats occurring throughout Fjii.

Except during low tides in calm weather, waves push water over the reef front, and water then flows laterally across the reef flats to discharge through channels bisecting the flats. This creates a relatively predictable current direction at most locations.

### Survival experiments

To test whether juvenile corals experienced distance-dependent mortality near adult conspecifics, we created ∼5 mm tall fragments of *P. damicornis* and *S. hystrix*, selected suitable adult focal colonies (defined below), attached conspecific fragments 3, 12, 24 and 182 cm up- and down-current from each focal adult, and monitored fragment survival. General direction of current (east to west) was determined by in-water observations over ∼7 years of working at this site. We conducted this experiment in Votua village’s MPA, which supports a diverse assemblage of corals covering about 50% of hard substrates (Rasher, Hoey & Hay, 2013). Water flow across this reef flat is often negligible around low tide, reducing the potential for constantly dissipating microbial enemies away from focal colonies.

We used pliers to clip 16 fragments of 30–40 polyps each from the tips of each of 24 large *P. damicornis* and 24 large *S. hystrix* colonies in the Votua village MPA. We employed fragments from older colonies as proxies for ∼6 month old juveniles (Sato, 1985) because, despite these species reproducing monthly in some locations (Fan et al., 2002; Kuanui et al., 2008), neither species planulated at our site during the months of this study (August through October 2013). The fragments from each of four source colonies for a species were collected in six rounds over two days. Each round was taken to shore and four fragments (one from each source colony) were epoxied (Emerkit epoxy) onto the unglazed side of 16 2.54 × 2.54 cm tiles. Thus, each tile had fragments from four different colonies and sets of 16 tiles had fragments from the same four colonies of the same species. After epoxying, tiles were held in a tub of seawater for ∼1 h, allowing the epoxy to harden. Tiles were then cable-tied onto metal racks at ∼1 m deep in the MPA and allowed to acclimate for two weeks before deployment in the experiment. Survivorship during acclimation was 100%, producing 384 fragments on 96 tiles for each coral species.

Within the MPA, 10 adult *P. damicornis* and 10 adult *S. hystrix* colonies served as focal colonies. Focal colonies: (i) were >10 cm at their smallest diameter (10 to 35 cm for *P. damicornis* and 10 to 75 cm for *S. hystrix*), (ii) had no conspecific colonies within 4 m (so as not to confound effects of the focal colony with effects of nearby conspecifics), (iii) were 5–40 cm deep at low tide, and (iv) had space for 190 cm PVC pipes to be placed roughly east and west (the predominant current direction was west) without disturbing other corals. Focal colonies were photographed from above and their size determined using ImageJ (Rasband, 1997).

**Figure 1:**
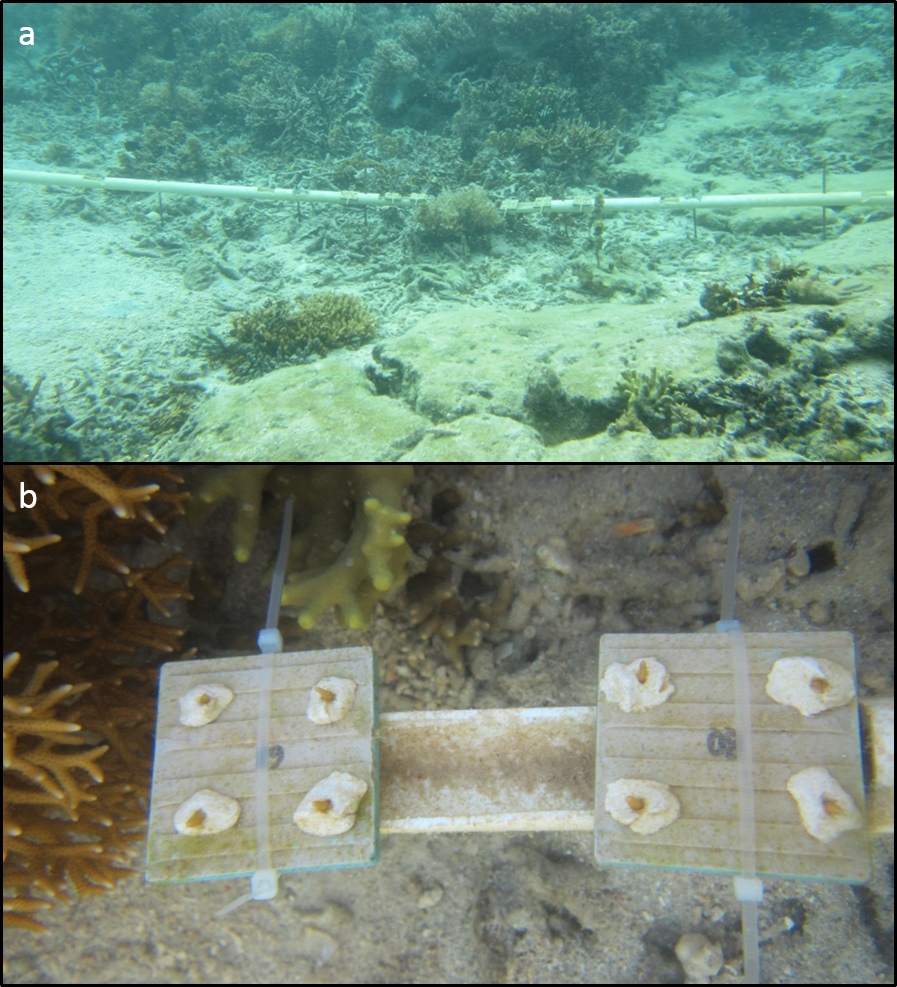
*Seriatopora hystrix* fragments on tiles on PVC array. Experimental set up of coral fragments epoxied to tiles on PVC pipes around focal adult colonies. (A) PVC pipes extending upstream and downstream from a focal *Seriatopora hystrix* colony. (B) Fragments epoxied onto ceramic tiles around a *S. hystrix* colony.

Twenty mm diameter by 190 cm long PVC pipes served as platforms to which we attached the tiles. Pipes were anchored to the reef by driving steel rebar through pre-drilled holes and cementing the rebar to the pipe. Notches 2.54 cm long allowed us to cable-tie tiles onto the pipes at distances of 3, 12, 24 and 182 cm from focal colonies (Figs. 1A and 1B). This approach secured all pipes and tiles throughout the experiment. These distances and this scale were chosen to match a previous experiment in the Caribbean that had detected distance-dependent mortality of newly settled recruits for a broadcast spawning coral (Marhaver et al., 2013).

Tiles were randomly assigned to positions on pipes. Thus, fragments at each distance and around each conspecific focal colony were random with respect to source colony. Unassigned tiles were kept on the rack as spares (64 fragments on 16 tiles for each coral species).

Every 1–2 d after deployment, we examined all fragments, recording survivorship, consumption, overgrowth by algae, bleaching, or other changes in status. The *P. damicornis* fragments were observed for 59 days. The *S. hystrix* fragments were deployed one month later and observed for 29 days.

On some *P. damicornis* tiles, three or four of the fragments disappeared within a 24 h period between checks on their condition, appearing to have been bitten off. To determine the agents of this localized mortality, we replaced tiles whose four fragments had been eaten with spare tiles holding four healthy fragments around three of the focal colonies that had experienced localized mortality and videotaped the tiles (GoPro II HD) from about 1 m away during the following high tides. Cameras were retrieved at the next low tide and the videos watched.

We evaluated overall survival patterns and mortality specifically due to bleaching or predation using mixed-effects Cox proportional hazards survival models (coxme package, Therneau, 2012) in R (R Core Team, 2014). Distance and direction from focal colony were fixed effects (4 levels and 2 levels, respectively) and focal colony and tile nested within focal colony were random effects because fragments were blocked by tile and focal colony. The size of the focal colony and the depth of the tiles were included as covariates. Finally, we compared the relative levels of predation and bleaching in *P. damicornis* and *S. hystrix* using a chi-square test.

### Distribution surveys

We characterized the spatial distribution of *P. damicornis* and *S. hystrix* in the reef flat MPAs of Namada, Vatu-o-lailai, and Votua villages at two scales (August through October 2013). For the larger-scale survey, we mapped each colony within 8 × 8 m plots (*N* = 5, 5, and 10 for Namada, Vatu-o-lailai, and Votua, respectively). Each plot was divided into 256 0.5 × 0.5 m cells and each coral ≥1 cm across mapped into a cell. The location of each survey plot was determined by randomly choosing a point on shore, swimming 100, 200, or 300 kicks directly away from shore at that point, and surveying the closest bommie (coral patch) large enough to fill more than three quarters of an 8 × 8 m plot. In four of 10 surveys at Votua and in all five surveys at Vatu-o-lailai and Namada, we also measured the largest diameter of each *P. damicornis* colony. We did not measure *S. hystrix* colony size because the frequent discontinuity of colonies made accurate estimation of colony area too error-prone. To avoid confounding biotically-driven spatial distribution with patterns caused by patchiness of suitable substrate, we also recorded which cells were comprised primarily of unsuitable habitats such as sand-scoured pools or channels and bommie tops covered in rubble.

We analyzed these data using the neighborhood density function O(r) in the point pattern analysis program Programita (Wiegand & Moloney, 2004). This analysis identifies distances at which individuals are aggregated, randomly spaced, or overdispersed compared to a specified null model. Unlike the more frequently used Ripley’s K(r) statistic, each distance category is not affected by those inside it; expected aggregation at each distance is compared to the observed value independently of nearer distances. Each concentric ring centered on an individual coral is separately placed on the aggregated-overdispersed continuum and displays the spatial pattern within a different distance category. Ring width was 0.5 m extending up to 4 m. The null model for this analysis was complete spatial randomness (CSR). Because the variance in substrate types violated CSR’s assumption of uniform likelihood of coral placement, we conducted the below analyses once using the entirety of all 8 × 8 m plots and a second time excluding cells of unsuitable habitat (which should better meet CSR’s assumption of uniform likelihood).

To determine whether the observed spatial pattern was random, significantly aggregated, or overdispersed, Programita simulated placement of each plot’s colonies 999 times using CSR, calculated O(r) for each simulation, then combined replicate O(r)’s from each reef and from all three reefs. This generated a distribution of simulated O(r)’s from which we established the significance of the observed spatial patterns. The distance(s) at which significant aggregation or overdispersion occurred were determined by the distances at which the observed pattern fell above or below the 95% simulation envelopes, respectively. This analysis does not parse aggregating and overdispering processes; it shows the net resulting pattern.

In addition to analyzing all *P. damicornis* and *S. hystrix* colonies, we analyzed *P. damicornis* <5 cm, ≥5 cm, ≥10 cm, and ≥15 cm in diameter to see if spatial patterns changed with colony size. The <5 cm and ≥5 cm categories were mutually exclusive but because there were not enough colonies between 5 and 10 cm and between 10 and 15 cm to analyze as mutually exclusive groups, larger size categories were subsets of smaller ones.

The 8 × 8 m quadrat surveys could not resolve spatial patterns below the cell size of 0.5 × 0.5 m, meaning that patterns occurring at less than 0.25^2^ m could be undocumented. To determine the spatial distribution of *P. damicornis* and *S. hystrix* at smaller scales, we conducted 2 m radius circular surveys around focal *P. damicornis* and *S. hystrix* colonies that (i) were the largest colony of that species within 4 m (to reduce the effects of conspecifics), and (ii) occurred where >75% of the substrate within 2 m was suitable habitat for *P. damicornis* and *S. hystrix*, again to equalize the likelihood of colonies occurring everywhere in the survey.

The distance to each surrounding (radial) *P. damicornis* and *S. hystrix* colony was the average of the distance to that colony’s near and far sides (*N* = 45 focal colonies for *P. damicornis* around *P. damicornis*, 10 for *S. hystrix* around *P. damicornis*, and 24 each for *P. damicornis* and *S. hystrix* around *S. hystrix*). We analyzed radial colony counts in 10 cm concentric rings using a generalized linear mixed effects model with Poisson errors and the canonical log link function in R (lme4 package, Bates et al., 2013). Distance was a fixed effect and focal colony was a random effect, with the log_10_ of the ring sizes as an offset to control for unequal area sampled at each distance (i.e., ring area increased with distance from the focal colony). We repeated this analysis with just the closest 0.5 m and 1 m of the circles in case radial colonies beyond those distances were masking short-range effects of the focal colonies.

We also analyzed the *P. damicornis* data from the 8 × 8 m plots in the same manner as we did the circular surveys. To convert the plot data, a function was written in R to identify every surveyed *P. damicornis* colony ≥2 m from all edges of its plot and equal to or larger than a specified diameter (either 15 or 20 cm) as a focal colony (*N* = 38 and 19 focal colonies, respectively). In order to have an appreciable sample size, we did not restrict focal colonies to those that were the largest within 4 m. The script then calculated the distances to all *P. damicornis* colonies less than the specified focal colony diameter within 2 m and placed them into 10 cm concentric rings as above. We analyzed the resulting data using the same procedures as described for the circular surveys.

## Results

### Survival experiments

**Figure 2:**
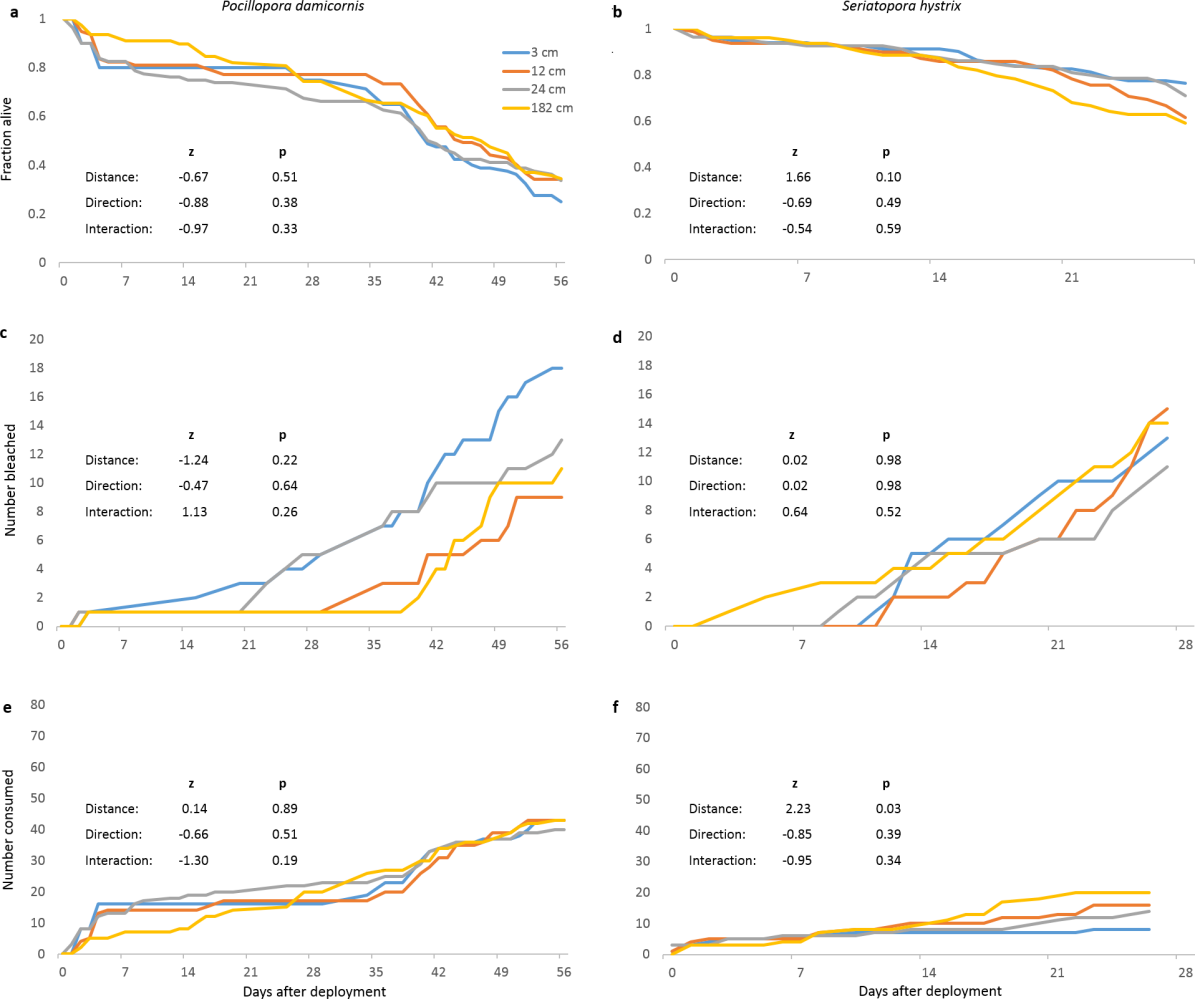
Survival and predation of *Pocillopora damicornis* and *Seriatopora hystrix*. (A), (C), (E) are *Pocillopora damicornis* and (B), (D), (F) are *Seriatopora hystrix*. Statistical values are from mixed-effects Cox proportional hazards survival analyses. *n* = 80 fragments at each distance across 10 focal colonies and pooled between both directions. (A, B) Survivorship through time for *Pocillopora damicornis* and *Seriatopora hystrix* fragments. (C, D) Cumulative number of fragments that bleached over time. (E, F) Cumulative number of fragments that partially or fully disappeared over time (putative predation).

In our field experiment, neither distance nor direction from focal colony significantly affected survival of *P. damicornis* or *S. hystrix* fragments (Figs. 2A and 2B, respectively). We observed two main categories of mortality: bleaching preceding death in place (potentially due to microbes (e.g., Ben-Haim, Zicherman-Keren & Rosenberg, 2003)) and partial or complete disappearance, putatively due to predation (akin to Lenihan et al., 2011). Bleaching (47 and 46 fragments out of 320 for *P. damicornis* and *S. hystrix*, respectively) of neither species was affected by distance or direction (Figs. 2C and 2D). Distance and direction did not affect the number of *P. damicornis* fragments that partially or fully disappeared (putative predation), and direction did not affect this for *S. hystrix* but distance was significant (*z* = 2.23, *p* = 0.03) (Figs. 2E and 2F), with disappearance increasing with distance from the focal colony. In contrast, 0% of the 64 extra fragments of each species remaining on the coral rack where we originally acclimated the corals bleached or disappeared despite being on the same reef at the same time (Cox proportional hazards survival analysis, likelihood ratio for *P. damicornis* = 16.5, likelihood ratio for *S. hystrix* = 24.7, *p* < 0.0001 for both species). Fragments on the coral rack were ∼1 m above the benthos and may have experienced more flow or fewer benthic-associated biotic or physical stressors compared to the fragments on PVC pipes, which were 5–15 cm above the benthos.

The rapid disappearance of *P. damicornis* fragments around some focal colonies suggested spatially localized predation. Therefore, we further divided deaths due to putative predation between isolated predation incidents (disappearance of one or two fragments on a tile in 24 h) and localized predation episodes (disappearance of three or four fragments from a tile in 24 h). We used this classification scheme because three or four fragments tended to disappear from multiple tiles within the same 24 h period at certain replicates, whereas the disappearance of one or two fragments was not often temporally coincident across tiles within a replicate. We distinguished between these two types of putative predation because their causes were potentially different and therefore either one could have been distance-dependent or masked distance-dependence in the other. Six of 10 *P. damicornis* replicates (23 out of 160 tiles) experienced localized predation on at least one of their eight tiles; three of those experienced localized predation on five or more tiles within 24 h. Two of 10 *S. hystrix* replicates experienced localized predation (on one tile each). We further investigated localized predation only for *P. damicornis* because localized predation on *S. hystrix* was infrequent.

When tiles that had experienced localized predation around three focal colonies were replaced with spare tiles holding healthy fragments, all three sets of replacement tiles again experienced localized predation and their collective survival was significantly lower than that of the replicates that did not experience localized predation in the initial run (mixed effect Cox proportional hazards, *z* = 3.5, *p* < 0.0005). Videos of these tiles showed the territorial triggerfish *Balistapus undulatus* consuming multiple fragments from multiple tiles around two of the three focal colonies. *Balistapus undulatus* feeding resulted in fragments irregularly broken at or above the top of the epoxy, as was seen for most localized predation episodes in the initial outplanting.

**Figure 3:**
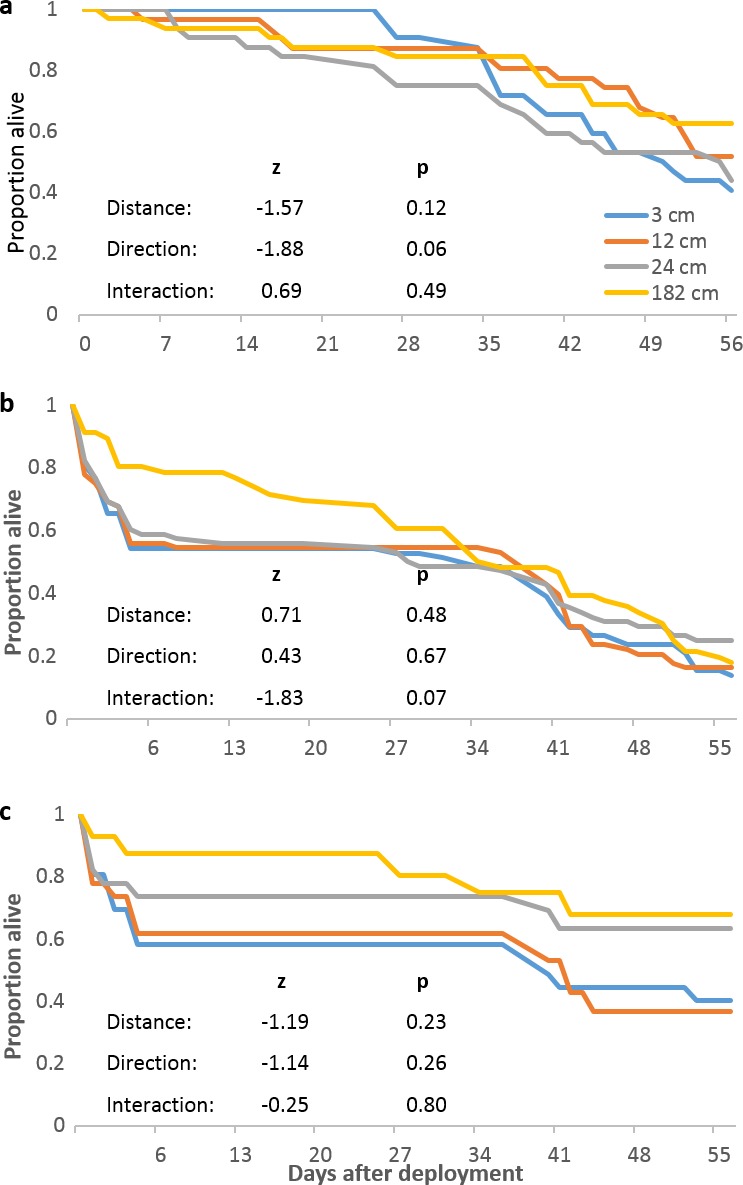
*Pocillopora damicornis* mortality relating to localized predation episodes. (A) Survival of *Pocillopora damicornis* fragments in replicates (4 focal colonies, 32 fragments at each distance) that did not experience localized predation. (B) Survival of *P. damicornis* fragments in the six focal colony replicates that did experience localized predation and in the replacement replicates. Deaths are from all causes. (C) Fraction of *P. damicornis* fragments not killed by localized predation episodes in original replicates that experienced localized predation and in the replacement replicates. Direction not shown. Analyses as in Fig. 2.

We next examined whether localized predation was distance-dependent and whether it masked distance-dependent mortality in replicates that did not experience localized predation. Distance and direction did not significantly affect mortality in replicates that did not experience localized predation (Fig. 3A). Considering only replicates that experienced localized predation (both original and replacement tiles), neither distance nor direction significantly affected mortality from all causes (Fig. 3B) or just from localized predation (Fig. 3C).

*Pocillopora damicornis* fragments were significantly more likely to die of putative predation as opposed to bleach and die in place than were *S. hystrix* fragments (chi-square test, *χ*^2^ = 17.2, *df* = 1, *p* < 0.0001). More than three times as many *P. damicornis* fragments died from putative predation as bleached prior to death (169 vs. 47 out of 320, respectively), while numbers of *S. hystrix* fragments that died from putative predation versus bleaching did not differ significantly (58 vs. 46 out of 320, respectively). Excluding replicates with localized predation, *P. damicornis* and *S. hystrix* appeared equally susceptible to isolated predation and bleaching (*χ*^2^ = 0.022, *df* = 1, *p* = 0.88).

### Distribution surveys

**Figure 4:**
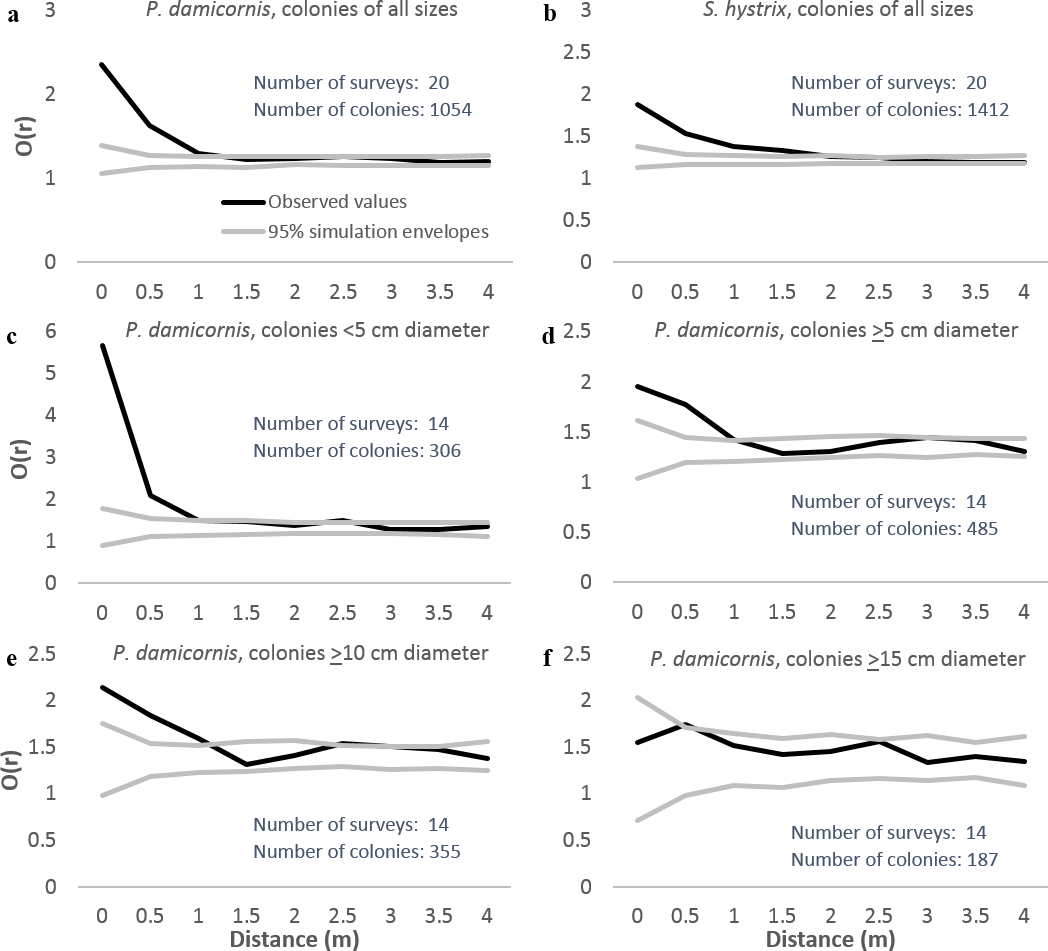
Neighborhood density analysis of *Pocillopora damicornis* and *Seriatopora hystrix* in 8 × 8 m quadrats with replicates from all three reefs combined. Black lines are observed patterns; grey lines are the 95% simulation envelopes from 999 simulations. Where black lines are above the upper grey line colonies are significantly aggregated, where they are between the grey lines colonies are randomly spaced, and where they are below the lower grey line colonies are significantly overdispersed. These analyses used only areas of suitable substrate (see text for definition).

We analyzed patterns of distribution using both entire 8 × 8 m plots and after excluding habitat deemed unsuitable for *P. damicornis* or *S. hystrix* (e.g., sand-scoured channels and pools, bommie tops covered in rubble). The analyses using only suitable habitat were quantitatively similar to those using the entire plots but were more conservative. Neighborhood density graphs using only suitable habitat are included here. Neighborhood density analysis indicated that both *P. damicornis* and *S. hystrix* were significantly aggregated at up to 1 m when all size classes were considered and surveys from all villages were pooled (Figs. 4A and 4B, respectively). When analyzed by site, the distance below which colonies were aggregated ranged from <1 m in Votua and Vatu-o-lailai to nearly 3 m in Namada. At no distance on any reef were colonies significantly overdispersed.

Identical analyses with *P. damicornis* separated into size categories (Figs. 4C–4F) indicated that the largest colonies (≥15 cm) were not aggregated at any scale, but all smaller size classes were strongly aggregated at scales of up to 1 m. Thus, smaller colonies appeared to drive the aggregation at up to ∼1 m when we analyzed all sizes together. However, the limited sample size for large colonies (*n* = 187) may have constrained our ability to detect spatial patterns for large colonies.

**Figure 5:**
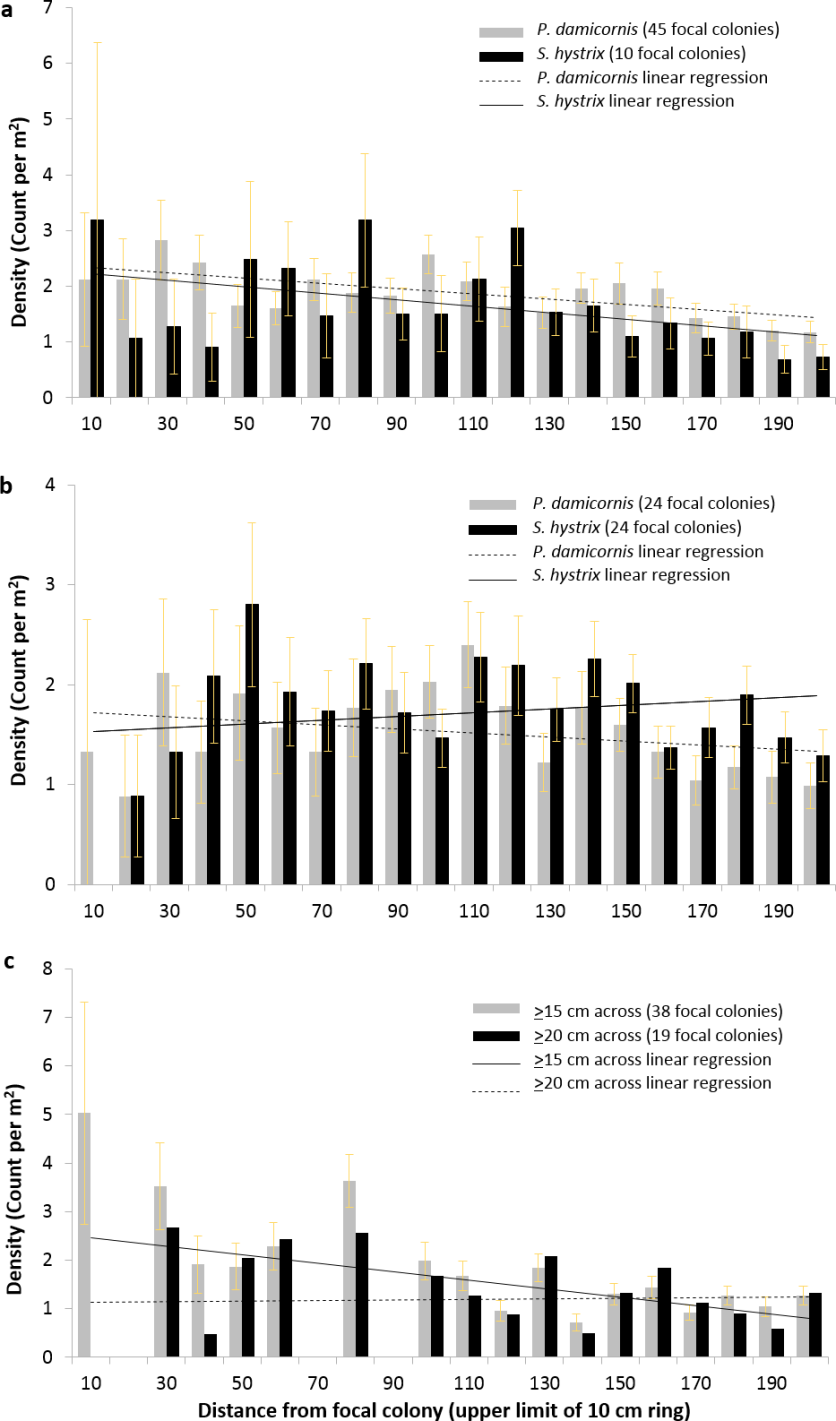
Density of *Pocillopora damicornis* and *Seriatopora hystrix* at 10 cm intervals around focal colonies. Density (±SE) of *Pocillopora damicornis* and *Seriatopora hystrix* at 10 cm intervals from focal (A) *P. damicornis* and (B) *S. hystrix* colonies. The linear regressions shown are to indicate the slope of the relationship found in the generalized linear mixed effects models but do not represent the models’ outputs. Radial colony count significantly declined with distance from focal colony over 2 m for three of the four focal-radial combinations (focal *P. damicornis*-radial *P. damicornis*—*z* = − 4.4, *p* < 0.001; focal *P. damicornis*-radial *S. hystrix*—*z* = − 3.9, *p* < 0.001; focal *S. hystrix*-radial *P. damicornis*—*z* = − 3.6, *p* < 0.001; focal *S. hystrix*-radial *S. hystrix*—*z* = − 1.9, *p* = 0.06). (C) Density (mean ±SE) of *Pocillopora damicornis* within 2 m of focal *P. damicornis* based on the 8 × 8 m surveys. Focal colonies are *P. damicornis* that are ≥15 cm across or ≥20 cm across. Radial colonies are any colonies below that size. Radial colony count significantly declined with distance over 2 m when colonies ≥15 cm were considered focal (focal colonies ≥15 cm—*z* = − 3.6, *p* < 0.0005; focal colonies ≥20 cm—*z* = − 1.09, *p* < 0.27).

To resolve the spatial distribution of *P. damicornis* and *S. hystrix* more finely, we conducted separate circular surveys (radius = 2 m) around focal colonies that met specific criteria. Across all 2 m, there was a significant negative relationship between distance from focal *P. damicornis* colonies and radial *P. damicornis* count (corrected for area surveyed at each distance and henceforth called density), focal *P. damicornis* and radial *S. hystrix* density, and focal *S. hystrix* and radial *P. damicornis* density (GLM: *z* = − 4.4, *p* < 0.0001; *z* = − 3.9, *p* < 0.0005; *z* = − 3.6, *p* < 0.0005, respectively) (Figs. 5A and 5B). The relationships within the first 0.5 m or 1 m for these focal-radial combinations were not significant (see Table 1 for all values not provided in text).

**Table 1:** Relationship between radial colony count and distance from focal colony. Relationship between count of radial *Pocillopora damicornis* and *Seriatopora hystrix* colonies and distance from focal *P. damicornis* and *S. hystrix* colonies using generalized linear mixed effects models. “Maximum distance” is the distance up to which radial colonies were considered.

Focal species–radial species	Maximum distance	Slope	*z* value	*p*-value
*P. damicornis*–*P. damicornis*	0.50 m	−0.0098	−1.0	0.32
	1.0 m	0.00055	0.22	0.83
	2.0 m	−0.0032	−4.4	<0.0001
*P. damicornis*–*S. hystrix*	0.50 m	0.016	0.59	0.56
	1.0 m	0.0012	0.19	0.85
	2.0 m	−0.0057	−3.9	<0.0005
*S. hystrix*–*P. damicornis*	0.50 m	0.0099	0.57	0.57
	1.0 m	0.0042	1.1	0.28
	2.0 m	−0.0036	−3.6	<0.0005
*S. hystrix*–*S. hystrix*	0.50 m	0.042	2.3	<0.05
	1.0 m	−0.00065	−0.18	0.86
	2.0 m	−0.0017	−1.9	0.06

Across all 2 m, there was no significant relationship between distance from focal *S. hystrix* colony and radial *S. hystrix* density (GLM, *z* = − 1.9, *p* = 0.06) (Fig. 5B). However, there was a significant positive relationship between distance and density within the first 0.5 m (GLM, *z* = − 12.99, *p* < 0.05) but not within the first 1 m.

When we converted the 8 × 8 m surveys into data analogous to the circular surveys and considered any *P. damicornis* colony ≥15 cm across as a focal colony and any smaller individual as a radial colony, there was a significant negative relationship between distance and radial *P. damicornis* density (GLM, *z* = − 3.6, *p* < 0.0005) across all 2 m but not across the first 0.5 m or 1 m (Fig. 5C and Table 2). However, when the cutoff for focal colonies was 20 cm, there was no relationship between distance and *P. damicornis* colony count at 0.5 m, 1 m, or 2 m (Fig. 5C and Table 2).

**Table 2:** 8 × 8 survey results converted to radial survey results. Relationship between count of radial *Pocillopora damicornis* colonies and distance from focal *Pocillopora damicornis* colonies using the data from the 8 × 8 m surveys. “Threshold size for focal colony” is the size above which surveyed colonies were designated “focal” and below which colonies were designated “radial.” “Maximum distance” is the distance up to which radial colonies were considered.

Threshold size for focal colony	Maximum distance	Slope	*z* value	*p*-value
15 cm	0.50 m	−0.0064	−0.46	0.65
	1.0 m	−0.0063	−1.5	0.14
	2.0 m	−0.0034	−3.6	<0.0005
20 cm	0.50 m	0.047	1.2	0.23
	1.0 m	0.00032	0.045	0.96
	2.0 m	−0.0016	−1.09	0.27

## Discussion

Using small portions of adult *P. damicornis* and *S. hystrix* colonies to represent ∼6 month old juveniles, we tested for distance-dependent survivorship as a function of proximity to adult conspecifics. Survival experiments with *P. damicornis* and *S. hystrix* fragments did not show distance-dependent mortality around conspecific adults (Figs. 2A and 2B). The lack of distance-dependent mortality in this study is consistent with a meta-analysis of distance-dependent mortality studies of the seeds and seedlings of terrestrial plants (Hyatt et al., 2003), in which distance from parents did not affect overall survival. However, when separated by life stage, that meta-analysis found that seedling survival increased with distance from parents while seed survival was not affected, suggesting that the strength of distance-dependent mortality may be a function of age. Our experiment using small coral fragments to represent juveniles attempted to conduct a similar test with corals. Our procedures would not have detected distance-dependent mortality of larvae occurring just after settlement. We would have preferred to conduct a reciprocal transplant experiment of fragments from both corals at differing distances to both conspecific and heterospecific adults but we were unable to gain permission to use that many coral colonies. Thus, we could document spatial patterns of survivorship relative to conspecific adults but not relative to heterospecific adults.

Spatial analyses of the distribution of conspecific colonies might uncover patterns that our short-term experiment could not detect. Observed spatial patterns represent the balance of multiple, potentially opposing processes, such as greater recruit density near brooding parents (similar to terrestrial seed shadows) versus detrimental effects of adult-associated enemies or intraspecific competition on aggregated, nearby recruits. Rather than overdispersion, we found significant clumping within 1 m of conspecifics for both *P. damicornis* and *S. hystrix* (Figs. 4A and 4B). The 8 × 8 m surveys and the 2 m radius surveys both supported this pattern; there was a significant negative relationship between *P. damicornis* radial colony density and distance from focal *P. damicornis* and a nearly significant negative relationship (with a much more limited sample size) between *S. hystrix* radial colony density and distance from focal *S. hystrix* (Figs. 5A and 5B). We also observed a significant negative relationship between *S. hystrix* density and distance from *P. damicornis* and *P. damicornis* density and distance from *S. hystrix* (Figs. 5A and 5B), suggesting that the cause of declining density with distance need not be species-specific. Since *P. damicornis* and *S. hystrix* are confamilial, it is possible that they aggregate because a location that is physiologically beneficial for one might also be beneficial for the other. We did detect one pattern consistent with the Janzen–Connell hypothesis: small colonies of *P. damicornis* were aggregated at scales of up to 1 m, while colonies ≥15 cm in diameter were not aggregated at any scale (Fig. 4). This selective loss of small colonies near adults is consistent with the Janzen–Connell hypothesis, but is also consistent with self thinning from intraspecific competition without mortality due to enemies aggregated near adults (Zhu et al., 2013).

There are a few potential causes for the observed clumping of conspecifics that could counteract Janzen–Connell effects (Carlon & Olson, 1993). Aggregated settlement near maternal adults may occur for *P. damicornis* and *S. hystrix* because brooded planulae can settle quickly after release (Richmond, 1987; Isomura & Nishihira, 2001; Underwood et al., 2007; Torda et al., 2013), and even if planulae disperse meters or kilometers, they may still aggregate near conspecific adults (Babcock, 1988; Tioho, Tokeshi & Nojima, 2001; Doropoulos et al., 2015). Moreover, pocilloporid recruitment is inherently spatially heterogeneous (Dunstan & Johnson, 1998) and occurs in hotspots that may be partially determined by water flow, density of adult confamilials (Eagle, 2006), and substrate suitability (Harriott, 1983; Lee, Walford & Goh, 2009). Thus, multiple ecologically important processes and interactions can generate aggregation of juveniles, and some of these could overwhelm Janzen–Connell effects and make them seem unimportant in the field (at least in the short term), even if they were occurring.

The only other direct test of the Janzen–Connell hypothesis in corals was conducted on planulae and recruits of broadcasting *Orbicella* (formerly *Montastraea*) *faveolata* in the Caribbean (Marhaver et al., 2013). In that study distance-dependent mortality appeared to be microbially mediated, with effects differing upstream and downstream of focal *O. faveolata*. The design of that study and ours differs in several ways.

First, Marhaver et al. (2013) used planulae and recruits a few days old in their distance-dependent survival experiments, whereas we used fragments taken from mature colonies. There are potential differences between fragments from adult corals and recruits. For example, the physiology, skeletal structure, and microbiomes of fragments from adults may differ from those of recruits and similarly sized juveniles (Vandermeulen & Watabe, 1973; Harriott, 1983; Le Tissier, 1988; Christiansen et al., 2009). The planulae of *P. damicornis* have higher lipid percentages in their tissue than do adult colonies (Figueiredo et al., 2012), which could affect palatability and microbial defense. Most recruit mortality in the study by Marhaver et al. (2013) appeared to be microbe-related as opposed to predator-generated. In contrast, predators generated considerable mortality of our juvenile sized transplants. Liberally assuming that every bleaching death in our study was due to microbes, only about one quarter of *P. damicornis* and half of *S. hystrix* fragments could have died directly because of microbes; thus, about 50–75% of the mortality we observed appeared to be due to consumption by fish. One way in which using colony fragments may not have been so different from using recruits was that both age categories may have similar zooxanthellae endosymbionts, since *P. damicornis* vertically transfers zooxanthellae to its young (LaJeunesse et al., 2004).

Second, Marhaver et al. (2013) studied the broadcast spawning species *O. faveolata*, while we studied two brooding species whose planulae may be more likely to settle near their parents, and whose larvae may receive critical components of their microbiome via vertical transmission from adults. Data on the make-up and function of juvenile coral microbiomes are limited but at present there is some evidence that larvae from brooding species may be more consistently endowed with parental components of the microbiome than are the larvae of broadcast spawners (Littman, Willis & Bourne, 2009; Apprill et al., 2012; Sharp, Distel & Paul, 2012; Lema, Willis & Bourne, 2012). In some acroporid corals, juveniles do not develop microbiomes typical of adult colonies until greater than 9 months of age (Littman, Willis & Bourne, 2009) but a core component of the microbiome appears in all the early stages, despite additions of other microbial species from the environment later in development (Lema, Willis & Bourne, 2012). However, in brooding species such as *Porites* and *Pocillopora*, critical microbes are transmitted from adults to larvae, or very quickly acquired from the environment, and even very young juvenile stages resemble adults in their composition of key microbes comprising the symbiotic microbiome (Apprill et al., 2012; Sharp, Distel & Paul, 2012). We do not know these relationships for the species we investigated but if their microbiomes take months to develop and are important defenses against microbial enemies, then our use of small adult portions may not mimic juvenile susceptibility to adult-associated pathogens. In contrast, if the critical components of the microbiome are present in even the earliest stages, then our adult fragments should be more representative. We would have preferred to use recently recruited larvae but neither *P. damicornis* nor *S. hystrix* planulated at our study site during our experiment.

Finally, the focal adult colonies of *Orbicella* investigated by Marhaver et al. (2013) form larger, longer-lived colonies than the colonies of *Pocillopora* and *Seriatopora* that we investigated. It is possible that larger, longer-lived colonies accumulate more species-specific enemies over their lifetimes; if so, this could more strongly suppress juvenile survivorship near these longer-lived adults.

Although we did not detect distance-dependent mortality, we did document spatially heterogeneous corallivory on *P. damicornis*. This may promote species coexistence by producing a mosaic of favorable and unfavorable patches for *P. damicornis* across the reef (Levin & Paine, 1974; Holt, 1984). Corallivore activity can structure coral distribution on reefs in both the Pacific and Caribbean (Neudecker, 1979; Littler, Taylor & Littler, 1989) and parrotfish and butterflyfish density can impact coral recruit and juvenile mortality, respectively (Penin et al., 2010). Localized predation by the triggerfish *Balistapus undulatus* on small *P. damicornis* could have a similar effect here. *Balistapus undulatus* is a generalist with territories of 100–200 m^2^ (McClanahan, 2000) and eats the tips of branching corals, including *P. damicornis* (Hiatt & Strasburg, 1960; Neudecker, 1979). This triggerfish species’ territoriality may delineate certain patches on reefs in which some species (e.g., *P. damicornis*) have high mortality while other species (e.g., *S. hystrix*) are not directly affected, akin to what is seen with seaweed in territories of the steephead parrotfish on the Great Barrier Reef (Welsh & Bellwood, 2012) or *Pocillopora* and *Pavona* in the interaction between damselfish territories and roving corallivores in the Eastern Pacific (Wellington, 1982). Additional experiments are necessary to determine how patchy corallivory contributes to the coexistence of *P. damicornis*, *S. hystrix*, and corals in general.

Overall we found little evidence for distance-dependent mortality relative to focal conspecific adults and for the pattern of over-dispersion that distance-dependent mortality would be expected to produce. Instead, both *P. damicornis* and *S. hystrix* aggregated at the scale of 1 m or less, with a tendency for small colonies to be clumped around larger ones. These findings suggest that local dispersal shadows or areas of physiological benefit near prospering adult conspecifics equal or exceed Janzen–Connell effects for the brooding corals we studied on these Fijian reef flats. Our experiments using small coral fragments did not detect distance-dependent mortality by species-specific enemies; we did, however, observe spatially heterogeneous corallivory on *P. damicornis*, which could facilitate species coexistence by delineating reef patches that are more or less favorable to different corals.

## Additional Information and Declarations

### Field Study Permissions

The following information was supplied relating to field study approvals (i.e., approving body and any reference numbers): Approval for our studies was granted by the Fijian Ministry of Education, National Heritage, Culture, and Arts, Youth, and Sports, and by the Korolevu-i-wai district environmental committee.

### Data Availability

The following information was supplied regarding data availability: Data in BCO-DMO database Project: Killer Seaweeds: Allelopathy against Fijian Corals http://www.bco-dmo.org/project/480717. For individual datasets:
http://www.bco-dmo.org/dataset/564411

http://www.bco-dmo.org/dataset/564397

http://www.bco-dmo.org/dataset/564389

http://www.bco-dmo.org/dataset/564404
http://www.bco-dmo.org/dataset/564418.

## References

[ref-1] Apprill A, Marlow HQ, Marntidale MQ, Rappe MS (2012). Specificity of associates between bacteria and the coral *Pocillopora meandrina* during early development. Applied Environmental Microbiology.

[ref-2] Babcock R (1988). Fine-scale spatial and temporal patterns in coral settlement.

[ref-3] Bagchi R, Gallery RE, Gripenberg S, Gurr SJ, Narayan L, Addis CE, Freckleton RP, Lewis OT (2014). Pathogens and insect herbivores drive rainforest plant diversity and composition. Nature.

[ref-4] Bates D, Maechler M, Bolker B, Walker S (2013). lme4: linear mixed-effects models using eigen and S4.

[ref-5] Ben-Haim Y, Zicherman-Keren M, Rosenberg E (2003). Temperature-regulated bleaching and lysis of the coral *Pocillopora damicornis* by the novel pathogen *Vibrio coralliilyticus*. Applied Environmental Microbiology.

[ref-6] Bernays EA (1989). Host range in phytophagous insects: the potential role of generalist predators. Evolutionary Ecology.

[ref-7] Bonaldo RM, Hay ME (2014). Seaweed-coral interactions: variance in seaweed allelopathy, coral susceptibility, and potential effects on coral resilience. PLoS ONE.

[ref-8] Buss LW, Jackson JBC (1979). Competitive networks: nontransitive competitive relationships in cryptic coral reef environments. American Naturalist.

[ref-9] Carlon DB, Olson RR (1993). Larval dispersal distance as an explanation for adult spatial pattern in two Caribbean reef corals. Journal of Experimental Marine Biology and Ecology.

[ref-10] Christiansen N, Ward S, Harii S, Tibbetts I (2009). Grazing by a small fish affects the early stages of a post-settlement stony coral. Coral Reefs.

[ref-11] Connell JH, Den Boer PJ, Gradwell GR (1971). On the role of natural enemies in preventing competitive exclusion in some marine animals and in rain forest trees.

[ref-12] Connell JH (1978). Diversity in tropical rain forests and coral reefs. Science.

[ref-13] Cornell JH, Karlson R (2000). Coral species richness: ecological versus biogeographical influences. Coral Reefs.

[ref-14] Dixson DL, Abrego D, Hay ME (2014). Chemically-mediated behavior of recruiting corals and fishes: a tipping point that may limit reef recovery. Science.

[ref-15] Doropoulos C, Ward S, Roff G, Gonzalez-Rivero M, Mumby PJ (2015). Linking demographic processes of juvenile corals to benthic recovery trajectories in two common reef habitats. PLoS ONE.

[ref-16] Dunstan P, Johnson C (1998). Spatio-temporal variation in coral recruitment at different scales on Heron Reef, southern Great Barrier Reef. Coral Reefs.

[ref-17] Eagle JV (2006). Recruitment hotspots around a coral reef: the roles of hydrodynamics and habitats. PhD Thesis.

[ref-18] Fan TY, Li JJ, Ie SX, Fang LS (2002). Lunar periodicity of larval release by pocilloporid corals in southern Taiwan. Zoological Studies.

[ref-19] Figueiredo J, Baird AH, Cohen MF, Flot J-F, Kamiki T, Meziane T, Tsuchiya M, Yamasaki H (2012). Ontogenetic change in the lipid and fatty acid composition of scleractinian coral larvae. Coral Reefs.

[ref-20] Fricke EC, Tewksbury JJ, Rogers HS (2014). Multiple natural enemies cause density-dependent mortality at the seed-to-seeding transition. Ecology Letters.

[ref-21] Harriott VJ (1983). Reproductive seasonality, settlement, and post-settlement mortality of *Pocillopora damicornis* (Linnaeus), at Lizard Island, Great Barrier Reef. Coral Reefs.

[ref-22] Hiatt RW, Strasburg DW (1960). Ecological relationships of the fish fauna on coral reefs of the Marshall Islands. Ecological Monographs.

[ref-23] Holt RD (1984). Spatial heterogeneity, indirect interactions, and the coexistence of prey species. American Naturalist.

[ref-24] Hyatt LA, Rosenberg MS, Howard TG, Bole G, Fang W, Anastasia J, Brown K, Grella R, Hinman K, Kurdziel JP (2003). The distance dependence prediction of the Janzen–Connell hypothesis: a meta-analysis. Oikos.

[ref-25] Isomura N, Nishihira M (2001). Size variation of planulae and its effect on the lifetime of planulae in three pocilloporid corals. Coral Reefs.

[ref-26] Janzen DH (1970). Herbivores and the number of tree species in tropical forests. American Naturalist.

[ref-27] Jayewardene D, Donahue MJ, Birkeland C (2009). Effects of frequent fish predation on corals in Hawaii. Coral Reefs.

[ref-28] Johnson DJ, Beaulieu WT, Bever JD, Clay K (2012). Conspecific negative density dependence and forest diversity. Science.

[ref-29] Jones G, Almany G, Russ G, Sale P, Steneck R, Van Oppen M, Willis B (2009). Larval retention and connectivity among populations of corals and reef fishes: history, advances and challenges. Coral Reefs.

[ref-30] Kuanui P, Chavanich S, Raksasab C, Viyakarn V (2008). Lunar periodicity of larval release and larval development of *Pocillopora damicornis* in Thailand. Marine & Freshwater Research.

[ref-31] LaJeunesse TC, Thornhall DJ, Cox EF, Stanton FG, Fitt WK, Schmidt GW (2004). High diversity and host specificity observed among symbiotic dinoflagellates in reef coral communities from Hawaii. Coral Reefs.

[ref-32] Lang J (1973). Interspecific aggression by scleractinian corals. 2. Why the race is not only to the swift. Bulletin of Marine Science.

[ref-33] Le Tissier MD’AA (1988). Patterns of formation and the ultrastructure of the larval skeleton of *Pocillopora damicornis*. Marine Biology.

[ref-34] Lee CS, Walford J, Goh BPL (2009). Adding coral rubble to substrata enhances settlement of *Pocillopora damicornis* larvae. Coral Reefs.

[ref-35] Lema KA, Willis BL, Bourne DG (2012). Corals form characteristic associations with symbiotic nitrogen-fixing bacteria. Applied Environmental Microbiology.

[ref-36] Lenihan HS, Holbrook SJ, Schmitt RJ, Brooks AJ (2011). Influence of corallivory, competition, and habitat structure on coral community shift. Ecology.

[ref-37] Levin SA, Paine RT (1974). Disturbance, patch formation, and community structure. Proceeding of the National Academy of Sciences of the United States of America.

[ref-38] Littler MM, Taylor PR, Littler DS (1989). Complex interactions in the control of coral zonation on a Caribbean reef flat. Oecologia.

[ref-39] Littman RA, Willis BL, Bourne DG (2009). Bacterial communities of juvenile corals infected with different S*ymbiodinium* (dinoflagellate) clades. Marine Ecology Progress Series.

[ref-40] Marhaver K, Vermeij M, Rohwer F, Sandin S (2013). Janzen–Connell effects in a broadcast-spawning Caribbean coral: distance-dependent survival of larvae and settlers. Ecology.

[ref-41] McClanahan T (2000). Recovery of a coral reef keystone predator, *Balistapus undulatus*, in East African marine parks. Biological Conservation.

[ref-42] Miller K, Mundy C (2003). Rapid settlement in broadcast spawning corals: implications for larval dispersal. Coral Reefs.

[ref-43] Nathan N, Casagrandi R (2004). A simple mechanistic model of seed dispersal, predation and plant establishment: Janzen–Connell and beyond. Journal of Ecology.

[ref-44] Neudecker S (1979). Effects of grazing and browsing fishes on the zonation of corals in Guam. Ecology.

[ref-45] Nozawa Y, Harrison PL (2008). Temporal patterns of larval settlement and survivorship of two broadcast-spawning acroporid corals. Marine Biology.

[ref-46] Packer A, Clay K (2000). Soil pathogens and spatial patterns of seedling mortality in a temperate tree. Nature.

[ref-47] Penin L, Michonneau F, Baird AH, Connolly SR, Pratchett MS, Kayal M, Adjeroud M (2010). Early post-settlement mortality and the structure of coral assemblages. Marine Ecology Progress Series.

[ref-48] Petermann JS, Fergus AJ, Turnbull LA, Schmid B (2008). Janzen–Connell effects are widespread and strong enough to maintain diversity in grasslands. Ecology.

[ref-49] Porter JW, Woodley JD, Jason Smith G, Neigel JE, Battey JF, Dallmeyer DG (1981). Population trends among Jamaican reef corals. Nature.

[ref-50] R Core Team (2014). R: a language and environment for statistical computing.

[ref-51] Rasband WS (1997). ImageJ, US National Institutes of Health.

[ref-52] Rasher DB, Hoey AS, Hay ME (2013). Consumer diversity interacts with prey defenses to drive ecosystem function. Ecology.

[ref-53] Richmond R (1987). Energetics, competency, and long-distance dispersal of planula larvae of the coral *Pocillopora damicornis*. Marine Biology.

[ref-54] Rotjan RD, Lewis SM (2008). Impact of coral predators on tropical reefs. Marine Ecology Progress Series.

[ref-55] Sato M (1985). Mortality and growth of juvenile coral *Pocillopora damicornis* (Linnaeus). Coral Reefs.

[ref-56] Sharp KH, Distel D, Paul VJ (2012). Diversity and dynamics of bacterial communities in early life stages of the Caribbean coral *Porites astreoides*. ISME Journal.

[ref-57] Therneau T (2012). coxme: mixed effects cox models.

[ref-58] Tioho H, Tokeshi M, Nojima S (2001). Experimental analysis of recruitment in a scleractinian coral at high latitude. Marine Ecology Progress Series.

[ref-59] Torda G, Lundgren P, Willis B, Oppen M (2013). Revisiting the connectivity puzzle of the common coral *Pocillopora damicornis*. Molecular Ecology.

[ref-60] Underwood J, Smith L, Van Oppen M, Gilmour J (2007). Multiple scales of genetic connectivity in a brooding coral on isolated reefs following catastrophic bleaching. Molecular Ecology.

[ref-61] Vandermeulen JH, Watabe N (1973). Studies on reef corals. I. Skeleton formation by newly settled planula larva of *Pocillopora damicornis*. Marine Biology.

[ref-62] Vermeij MJ (2005). Substrate composition and adult distribution determine recruitment patterns in a Caribbean brooding coral. Marine Ecology Progress Series.

[ref-63] Vermeij MJ, Sandin SA (2008). Density-dependent settlement and mortality structure the earliest life phases of a coral population. Ecology.

[ref-64] Wellington GM (1982). Depth zonation of corals in the Gulf of Panama: control and facilitation by resident reef fishes. Ecological Monographs.

[ref-65] Welsh J, Bellwood D (2012). Spatial ecology of the steephead parrotfish (*Chlorurus microrhinos*): an evaluation using acoustic telemetry. Coral Reefs.

[ref-66] Wiegand T, Moloney AK (2004). Rings, circles, and null-models for point pattern analysis in ecology. Oikos.

[ref-67] Zhu Y, Getzin S, Wiegand T, Ren H, Ma K (2013). The relative importance of Janzen–Connell effects in influencing the spatial patterns at the Gutianshan subtropical forest. PLoS ONE.

